# A Role of Socioeconomic Status in Cognitive Impairment Among Older Adults in Macau: A Decomposition Approach

**DOI:** 10.3389/fnagi.2022.804307

**Published:** 2022-02-08

**Authors:** Zhuo Zhang, Yonghua Zhao, Ying Bian

**Affiliations:** ^1^State Key Laboratory of Quality Research in Chinese Medicine, Institute of Chinese Medical Sciences, University of Macau, Taipa, Macau SAR, China; ^2^School of Health Services Management, Xi’an Medical University, Xi’an, China

**Keywords:** cognitive impairment, inequality, concentration index, Macau elderly, socioeconomic status

## Abstract

**Background:**

To explore the prevalence and inequality of cognitive impairment among older adults in Macau and the contributions of influencing factors to inequality in cognitive impairment.

**Methods:**

The Montreal Cognitive Assessment (MoCA) was used to measure the prevalence of cognitive impairment. Socioeconomic status scores were used to describe economic level of the subjects. The concentration index was applied to measure the inequality of cognitive impairment associated with socioeconomic status (SES) and decomposed into its influencing factors.

**Results:**

The prevalence of cognitive impairment was 44.35% in Macau. The concentration index of cognitive impairment among the elderly was −0.165 (95% CI: −0.232 to −0.098), indicating that the prevalence of cognitive impairment was concentrated in households with poor SES. Older age, poor SES, long or short sleep duration, other marital status, poor appetite, no tea/coffee drinking, no religious belief, and depression contributed positively to the inequality of cognitive impairment. Among these factors, SES made the largest contribution to inequality, accounting for 76.74%.

**Conclusion:**

In Macau, the prevalence of cognitive impairment among people aged 65 years and older is concentrated among those who are in poor economic conditions. Reducing the gap between the rich and the poor at the source, increasing educational level of low SES population and improving their access to healthcare services will help to improve the inequity of cognitive impairment.

## Introduction

With increasing life expectancy and declining fertility, China has experienced rapid aging over the past few decades, and at a significantly faster rate than most countries (Fang et al., 2020). There are 264.02 million people in China aged 60 years and above, accounting for 18.70% the total population, by the end of 2020 (Tang et al., 2021). As a result, the aging population is challenging the social security and health care systems. The aging population has led to a higher prevalence of chronic diseases and dysfunctions among older adults, with more than 100 million older adults in China suffering from chronic non-communicable diseases in 2013 (Wang and Chen, 2014). At the same time, more than 37 million older people have physical impairments (Miao et al., 2019). Cognitive impairment, as a chronic non-communicable disease, is becoming an important public health problem in China. Cognitive impairment (Cannon et al., 2017) refers broadly to various degrees of cognitive impairment from various causes, ranging from mild cognitive impairment (MCI) to dementia. Dementia is the most severe stage of cognitive impairment and is the leading cause of disability in people over 60 years of age worldwide, including China (Clay et al., 2019).

For families of people with cognitive impairment, it has been found that many of the caregivers reduced even basic requirements such as the food and medical care for them and their families to afford the high cost of medical care for people with dementia. The tremendous amount of cost associated with caring for someone with dementia puts millions of families below the poverty line (Luo et al., 2021). A study based on the global burden of disease from 1990 to 2019 showed that the number of new cases of AD and other dementias in China reached 1.803 million in 2019, with a disability loss of 2.223 million person-years. From 1990 to 2019, the disability-adjusted life year (DALY) values and DALY rates increased by 223.78 and 169.46%, respectively, with Years of Life Lost (YLL) contributing to the main burden of disease and premature death caused by disease being the main cause of the disease burden (Huang et al., 2021). The annual economic expenditure for AD patients nationwide is approximately 1.47% of the current year’s GDP, while the global cost of AD is 1.09% of the global GDP, so the disease burden of AD is higher in China than the world average in terms of economic burden. The total annual cost of AD is estimated to reach US$1.89 trillion in 2050 (Xu et al., 2017). In 2016, the Chinese government released the “Health China 2030 Strategic Planning Outline,” which states that the government will prioritize the health of the population. Minimizing the prevalence of cognitive impairment and incidence of disabling conditions in the elderly population at the source and enabling older adults with cognitive disorders to maintain a healthy and independent life for as long as possible are important measures to implement the national strategy to actively respond to population aging.

MCI (Langa and Levine, 2014) is defined as a decrease in memory or other cognitive function that does not affect the ability to perform activities of daily living and does not meet the diagnostic criteria for dementia compared to age and education matched normal older adults. Studies have noted that MCI, a prodromal stage of dementia, has 10 times the risk of progression to dementia than normal middle-aged and older adults (Sanford, 2017). Therefore, MCI-related research has received increasing attention as a breakthrough in dementia prevention and treatment. Health equity is one of the important indicators for evaluating health policies, that is, people with different incomes should have the same or similar levels of health. In health systems, health equity requires that all members of society have a fair chance to obtain the highest possible level of health (Gaffney and McCormick, 2017). Socioeconomic status (SES) is defined as a person’s combined economic and social status and is a composite indicator of an individual’s social status (Poulain et al., 2019). Socioeconomic differences in the prevalence of chronic diseases have been found worldwide (Zhang Y.B. et al., 2021).

Social stratification of health is prevalent in almost all societies, with higher socioeconomic status groups having better health on average than lower socioeconomic status groups. A number of studies have confirmed that the prevalence is significantly higher in people with lower socioeconomic and employment status (Wallis et al., 2002; Dalstra et al., 2005; Lai et al., 2019). Because cognitive impairment in the elderly is a complex multifactorial disease, the risk of its development is determined by a combination of genetic and lifelong environmental factors and their interactions. No drugs have been developed to effectively treat or slow the progression of cognitive impairment (Livingston et al., 2017). Moreover, symptoms in older adults are often overlooked because most of them mistakenly believe that cognitive impairment is part of the normal aging process. Notably, China has the highest number of people with dementia in the world, accounting for a quarter of the world’s total dementia population (Jia et al., 2020). Being a special segment of the population, health care for the elderly has always occupied an important place in China’s public health service system. Few studies have evaluated the extent of health inequalities and factors influencing cognitive impairment in Chinese populations. Therefore, this study analyses inequalities in cognitive impairment among older adults to provide population data evidence for researchers and policy makers for decision-making, using a representative sample of people aged 65 years and older in Macau. At the same time, it positively responds to the national strategy of aging in China and the long-standing goal of health equity shared by all human beings.

## Materials and Methods

### Study Design and Subjects

This study is a cross-sectional study conducted in Macau from September to December 2019. With the aging of Macau society, the proportion of people with cognitive impairment is increasing year by year. Cognitive impairment has become one of the most important public health problems affecting the quality of life of older people in Macau. This project aims to systematically explore the association of cognitive impairment with SES, lifestyle, and traditional Chinese Medicine constitution in Macau elders, and to examine intestinal flora changes, biometabolic pathways, and neurological imaging and behavioral assessments. Related studies have been previously reported (Zhang Z. et al., 2021). In this study, stratified random sampling was used to obtain a representative sample based on administrative structure. First, Macau was divided into three parts (Peninsula, Coloane and Taipa) and one elderly health center was randomly selected from each administrative region. Finally, Pou Tai Elderly Service Integrated Center, Areia Preta Minghui Nursing Home, and Enhui Elderly Service Integrated Center from the Taipa, Peninsula and Coloane, respectively, were selected for this survey. Secondly, 400 participants were randomly selected from those aged 65 years and above registered in these three elderly health centers who met the inclusion criteria. A total of 345 elderly people were finally included in this study because incomplete information (*n* = 55) was excluded. The selection criteria for the study population were: (1) 65 years of age or older; (2) Chinese residents of Macau; and (3) no major illnesses in the past year. Participants with heart failure, mental illness, tumors, and other serious systemic diseases that prevented them from completing the questionnaire even with assistance were excluded; (4) No language communication impairment, and able to understand and answer questions in Cantonese. All investigators underwent uniform training. Investigators were university or graduate students with medical backgrounds and fluent Cantonese communication skills. All participants were informed of the purpose, meaning, time requirements, and ethical principles of the study, and gave formal written consent before the study began. The investigator then conducted individual face-to-face interviews with each participant. We had calculated the sample size prior to the survey according to the following formula.


$$\text{N}=\frac{z^{2}\,p\,\left(1-p\right)}{e^{2}}$$

A sample size of 269 was required for this study at the maximum response distribution rate (*p* = 0.5, e = 0.1p) and 90% confidence level (*z* = 1.64), and we have expanded the sample size to 400 considering the non-response rate. This study ultimately included 345 respondents, which met the sample size requirement.

### Study Variables

In this study, cognitive impairment was used as the dependent variable and socioeconomic factors were used as independent variables. Participants’ cognitive functioning was assessed by the Montreal Cognitive Assessment-Hongkong (MoCA-HK) (Pan et al., 2020). The MoCA-HK has high diagnostic accuracy as a screening tool to identify MCI in Chinese older adults and is a valid and reliable tool for Chinese older adults in Hong Kong and Macau (Wong et al., 2009). Compared to other scales in its category, the MoCA-HK scale is brief (can be completed in 15 min), which is suitable for measurement needs in different settings (e.g., clinical settings). In addition, it avoids the ceiling effect in the use of the Mini-Mental State Exam (MMSE) (Shim et al., 2017) and the need to purchase it to use it. The scale includes cognitive dimensions such as visuospatial ability, naming, attention, language, abstract reasoning, memory, and orientation to time and place. The scale consists of 30 items. The higher the score, the better the neurocognitive function. The MoCA scale has a full score of 30, with ≥ 21 being normal. If the number of years of education is less than 6 years, 1 point is added to the result. Socioeconomic factors included education level, occupation, and economic status. Education level was divided into three groups: illiterate, elementary school and below, and junior high school and above, and was measured based on the highest education level self-reported by the participants. Occupation was divided into three groups: professional and technical, service, and industrial workers, and self-employed and family workers. Economic status was divided into four groups based on household income.

### Covariates

Covariates included demographic factors such as gender (male, female), age (< 75 years, ≥ 75 years), religion (none, yes), marital status (other, married), and lifestyle factors such as sleep duration (< 7 h, 7–9 h, ≥ 9 h), regular exercise (none, yes), appetite (bad, moderate, good), and tea/coffee consumption (none, yes), and psychological factors such as depression (none, yes). Among these, we used the Geriatric Depression Scale (GDS-15) to assess the level of mental health (Guerin et al., 2018). The total score is 15, and the higher the score, the more pronounced the depressive symptoms. The scale has 15 items that are answered with “yes” or “no.” A total score greater than 5 indicates the possibility of depression. Sleep duration was stratified based on the joint consensus statement of the American Academy of Sleep Medicine and the Sleep Research Society on the recommended amount of sleep (Watson et al., 2015; Grandner et al., 2018).

### Statistical Analysis

The analysis strategies of this study were as follows: Firstly, the basic characteristics of the subjects were described, the qualitative variables were described by frequency (*n*) and percentage (%), and the inter-group comparison was performed by χ^2^-test. Mean ($\bar{x}$) and standard deviation (SD) were used to describe quantitative variables. Secondly, the generalized linear model (GLM, distribution: binomial distribution, link function: Logit) was used to analyze the factors affecting the cognitive impairment. Thirdly, principal component analysis was used to calculate the SES score, and concentration index was used to analyze the inequality of cognitive impairment. To further verify the stability of the results, we stratified by gender to analyze the equity of cognitive impairment in older adults. STATA 16.0 was used to complete all statistical analysis processes in this study, and all tests were two-sided tests, a = 0.05.

#### Construction of Socioeconomic Status

SES was a commonly used indicator to describe family economic level, which comprehensively described the socioeconomic status of the subjects. SES generally included three dimensions: rank, identity and power (Galobardes et al., 2006). Combined with the actual situation of this survey and prior study (Wani, 2019), three indicators including education level, income and occupation were adopted to reflect the SES of the subjects. The score of SES was calculated by principal component analysis. The score of each index in the first principal component was used as the corresponding weight to calculate the score of SES. The formula of SES was as follows:


$$\left\{ \begin{array}{c} y_{1}=a_{11}\,x_{1}+a_{12}\,x_{2}+\ldots +a_{1\,j}\,x_{j}\\ y_{2}=a_{21}\,x_{1}+a_{22}\,x_{2}+\ldots +a_{2\,j}\,x_{j}\\ \vdots \;\\ y_{i}=a_{i\,1}\,x_{1}+a_{i\,2}\,x_{2}+\ldots +a_{i\,j}\,x_{j} \end{array}\right.$$

Where, *y*_*i*_ represented the principal component *i*, *x*_*j*_ represented the original index *j, a*_*ij*_ represented the coefficient. In this study, according to the third quantile, the SES was divided into three groups: poor, medium and good.

#### Concentration Index

Concentration index was an indicator reflecting the inequity of health status caused by social and economic factors. The reliability of using concentration index and its decomposition to measure health inequality had been fully verified in previous studies (Kakwani et al., 1997). This study used concentration index to measure the inequality of cognitive impairment among people of different SES. The value range of the concentration index was [−1, 1]. If people of different economic levels had the same probability of developing cognitive impairment, the concentration index was 0; if the concentration index was negative, it indicated that the prevalence of cognitive impairment tended to be poor; if the concentration index was positive, it indicated that the prevalence of cognitive impairment tends to be rich. The calculation formula was as follows:


$$\text{c}=\frac{2}{\mu }\,c\,o\,v\,\left(y_{i},R_{i}\right)$$

Where, *R*_*i*_ represented the proportion of individual *i* ranked by SES; *y*_*i*_ represented the prevalence of cognitive impairment; μ represented the average prevalence of cognitive impairment.

The concentration index decomposition method can decompose the concentration index into the contribution of influencing factors (Kavosi et al., 2012). The linear regression model was as follows:


$$y_{i}=a+\sum_{k}\beta_{k}\,x_{k_{i}}+\varepsilon_{i}$$

Where, β_*k*_ represented the regression coefficient, ε represented the residual. The concentration index can be decomposed by the following formula:


$$C=\sum_{K}\left(\beta_{k}\,{\bar{x}}_{k}/\mu \right)\,C_{k}+G\,C_{\varepsilon }/\mu$$

Where, β_*k*_, *C*_*k*_, $\beta_{k}\,{\bar{x}}_{k}/\mu$, $\left(\beta_{k}\,{\bar{x}}_{k}/\mu \right)\,C_{k}$ represented the regression coefficient, concentration index, Elasticity and contribution, respectively.

## Results

### Participant Characteristics

A total of 345 subjects were investigated in this study, and the prevalence of cognitive impairment was 44.35%. The prevalence of cognitive impairment in poor, medium and good SES groups was 58.97, 45.69 and 27.68%, respectively. Among them, 17.39% were males with an average age of 77.15 ± 8.22 years. Those who sleep > 9 and < 7 h accounted for 31.01 and 23.77%, respectively. 77.68% of the subjects took regular exercise, 57.97% were married, 8.41% reported bad appetite, 56.52% drank tea/coffee regularly, 38.55% had religious belief and 24.93% were depressed. Other information about the research object was shown in Table 1. The basic characteristics of the study population of each senior center were shown in Supplementary Table 1.

**TABLE 1:** Sociodemographic characteristics of subjects.

Variables	Total	SES		
		Poor (117)	Medium (116)	Good (112)
**Gender**				
Male	60 (17.39%)	8 (13.33%)	24 (40.00%)	28 (46.67%)*
Female	285 (82.61%)	109 (38.25%)	92 (32.28%)	84 (29.47%)
**Age (years)**				
<75	138 (40.00%)	29 (21.01%)	55 (39.86%)	54 (39.13%)*
≥ 75	207 (60.00%)	88 (42.51%)	61 (29.47%)	58 (28.02%)
**Sleep duration(hours)**				
<7	82 (23.77%)	34 (41.46%)	24 (29.27%)	24 (29.27%)
7–9	156 (45.22%)	41 (26.28%)	55 (35.26%)	60 (38.46%)
≥ 9	107 (31.01%)	42 (39.25%)	37 (34.58%)	28 (26.17%)
**Regular exercise**				
No	77 (22.32%)	23 (29.87%)	24 (31.17%)	30 (38.96%)
Yes	268 (77.68%)	94 (35.07%)	92 (34.33%)	82 (30.60%)
**Marital status**				
Others	145 (42.03%)	62 (42.76%)	50 (34.48%)	33 (22.76%)*
Married	200 (57.97%)	55 (27.50%)	66 (33.00%)	79 (39.50%)
**Appetite**				
Bad	29 (8.41%)	16 (55.17%)	10 (34.48%)	3 (10.34%)*
Medium	33 (9.57%)	13 (39.39%)	14 (42.42%)	6 (18.18%)
Good	283 (82.03%)	88 (31.10%)	92 (32.51%)	103 (36.40%)
**Tea/coffee drinking**				
No	150 (43.48%)	65 (43.33%)	47 (31.33%)	38 (25.33%)*
Yes	195 (56.52%)	52 (26.67%)	69 (35.38%)	74 (37.95%)
**Religious belief**				
No	212 (61.45%)	71 (33.49%)	74 (34.91%)	67 (31.60%)
Yes	133 (38.55%)	46 (34.59%)	42 (31.58%)	45 (33.83%)
**Depression**				
No	259 (75.07%)	84 (32.43%)	88 (33.98%)	87 (33.59%)
Yes	86 (24.93%)	33 (38.37%)	28 (32.56%)	25 (29.07%)
**Cognitive impairment**				
No	192 (55.65%)	48 (41.03%)	63 (54.31%)	81 (72.32%)*
Yes	153 (44.35%)	69 (58.97%)	53 (45.69%)	31 (27.68%)

*P < 0.05; SES, socioeconomic status.

### Analysis of Factors Affecting Cognitive Impairment

Univariate analysis showed that the prevalence of cognitive impairment was different among different SES, sleep time, marital status, appetite, tea/coffee drinking and depression groups (Supplementary Table 2). GLM show that good SES (OR = 0.31, 95%CI: 0.17∼0.58), married (OR = 0.60, 95%CI: 0.36∼0.99) reduced the risk of cognitive impairment and sleep duration > 9 h (OR = 1.75, 95%CI: 1.01∼3.04), depression (OR = 1.76, 95%CI: 1.02∼3.04) increased the risk of cognitive impairment (Table 2).

**TABLE 2:** Regression analysis results for cognitive impairment.

	OR	95%CI	*P*
**Gender**			
Female	0.83	0.43∼1.61	0.582
**Age (year)**			
≥ 75	1.00	0.61∼1.65	0.997
**SES**			
Medium	0.60	0.34∼1.07	0.084
Good	0.31	0.17∼0.58	<0.001
**Sleep duration (h)**			
<7	0.76	0.42∼1.39	0.376
≥ 9	1.75	1.01∼3.04	0.045
**Regular exercise**			
Yes	0.74	0.42∼1.30	0.293
**Marital status**			
Married	0.60	0.36∼0.99	0.046
**Appetite**			
Medium	2.40	0.78∼7.37	0.127
Good	0.97	0.41∼2.28	0.940
**Tea/coffee drinking**			
Yes	0.68	0.42∼1.09	0.108
**Religious belief**			
Yes	0.77	0.47∼1.24	0.284
**Depression**			
Yes	1.76	1.02∼3.04	0.041

*SES, socioeconomic status.*

### Inequity Analysis of Cognitive Impairment

**FIGURE 1:**
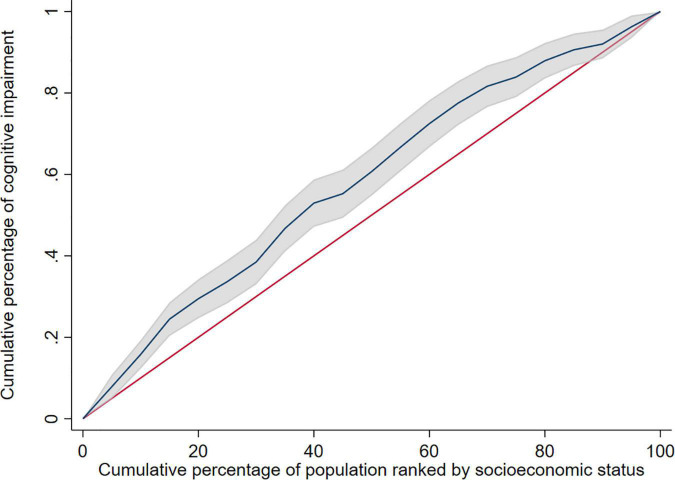
Concentration curve of cognitive impairment. The shadow around the curve represent 95%CI.

The study used education level, income, and occupation to describe SES, and the first principal component of each indicator were 0.557∼0.605 (Table 3). The concentration index of cognitive impairment in Macao elderly was −0.165 (95%CI: −0.232 to −0.098), indicating that the prevalence of cognitive impairment had a certain degree of inequality, and the prevalence of cognitive impairment was mainly concentrated in subjects with poor SES (Table 4). Figure 1 shows the concentration curve, which is above the reference line. The concentration index calculated from the SES dimensions was −0.134 (95%CI: −0.195 to −0.073) (education level), −0.053 (95%CI: −0.053 to 0.010) (income) and −0.140 (95%CI: −0.199 to −0.081) (occupation), respectively. In this study, logit model was established to further decompose the concentration index and to determine the contribution rate of each research factors. The results showed that older age, poor socioeconomic status, long or short sleep duration, other marital status, poor appetite, no tea/coffee drinking, no religious belief, and depression contributed positively to the inequality of cognitive impairment. Among these factors, socioeconomic status contributed the most to the inequality, accounting for 76.74%. Marriage and appetite contributed 8.63 and 5.52%, respectively (Table 5).

**TABLE 3:** First principal component weights of variables related to SES.

Variables	First principal component
Education level	0.557
Income	0.569
Occupation	0.605

**TABLE 4:** Concentration index of cognitive impairment in the elderly under different socioeconomic status indicators.

Indicator	Concentration index	95%CI	*P*
SES	−0.165	−0.232∼−0.098	<0.001
**Dimensions**			
Education level	−0.134	−0.195∼−0.073	<0.001
Income	−0.053	−0.053∼0.010	0.102
Occupation	−0.140	−0.199∼−0.081	<0.001

**TABLE 5:** Decomposition analysis on the inequality of cognitive impairment.

	Elasticity	Concentration index	Contribution	Contribution rate
Gender				−2.38%
Male	0.073	0.247	0.018	
Age (years)				0.02%
≥ 75	0.001	–0.109	<0.000	
SES				76.74%
Poor	0.889	–0.663	–0.589	
Medium	0.499	0.015	0.007	
Sleep duration (h)				3.50%
<7	–0.145	–0.068	0.010	
≥ 9	0.393	–0.093	–0.036	
Regular exercise				−1.50%
Yes	–0.530	–0.021	0.011	
Marital status				8.63%
Others	0.483	–0.135	–0.065	
Appetite				5.52%
Bad	0.006	–0.291	–0.002	
Medium	0.196	–0.205	–0.040	
Tea/coffee drinking				5.19%
No	0.381	–0.103	–0.039	
Religious belief				0.67%
No	0.366	–0.014	–0.005	
Depression				3.60%
Yes	0.319	–0.086	–0.027	

*SES, socioeconomic status.*

### Stratified Analysis

**FIGURE 2:**
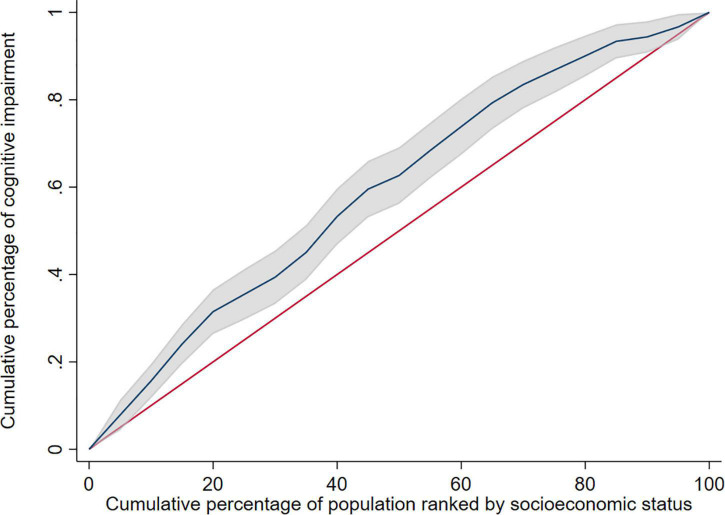
Concentration curve of cognitive impairment in female elderly. The shadow around the curve represent 95%CI.

Stratified analysis based on gender revealed that the concentration index of cognitive impairment was −0.024 (95% CI: −0.110 to 0.062) in male elderly and −0.191 (95% CI: −0.262 to −0.120) in female elderly. The concentration index was negative in both male elderly and female elderly, but the concentration index was not statistically significant in male elderly, and the inequity of cognitive impairment was more severe in female elderly than male elderly. The decomposition analysis of the concentration index and concentration curve were shown in Supplementary Table 3 and Figure 2, respectively.

## Discussion

Cognitive impairment is one of the leading causes of disability and dependency in older adults worldwide. Cognitive impairment has physical, psychological, social, and economic effects on the patients themselves, as well as on their caregivers, families and society at large. This study measured the prevalence of cognitive impairment among older adults aged 65 years and older in Macau using data from a representative sample in Macau. The results of the study found that the prevalence of cognitive impairment among older adults in Macau was 44.35%. The higher prevalence of cognitive impairment in the present study compared to other studies may be due to the older age of the study population. In addition, the prevalence of cognitive impairment differs between domestic and international studies due to the different survey instruments, diagnostic criteria, and populations studied (Lopez et al., 2003; Kim et al., 2011). The study also analyzed the factors influencing cognitive impairment and found that poor socioeconomic status, long sleep duration (> 9 h), depression, and unmarried status increased the risk of cognitive impairment. This is generally consistent with the findings of previous studies (Yang et al., 2016).

In this study, education level, income, and occupation were used to describe socioeconomic status. It is widely accepted that low SES is one of the risk factors for cognitive impairment in older adults (Kelly et al., 2016). The mechanisms by which SES affects cognitive impairment are not fully understood, but it is hypothesized that SES may mitigate cognitive impairment by building and preserving brain reserve capacity. The “cognitive reserve” hypothesis refers to the effect of intellectual stimulus-related factors (e.g., education, occupation, leisure intellectually stimulating activities, and social interactions) on brain tissue pathological load manifested as cognitive dysfunction. Those with higher cognitive reserve may delay the onset of cognitive impairment or dementia symptoms caused by brain pathological changes through some compensatory mechanism (Cermakova et al., 2018). People with low SES have low health literacy because of their low level of education. Also, they are less likely to receive health advice and have less initiative to be screened for cognitive impairment compared to those with high SES, combined with limited access to health resources, and their economic status leads to limited social participation in low SES people, who do not have enough time and energy to enrich social activities and engage in different forms of psychosocially stimulating activities to expand their cognitive reserve and thus buffer the risk of cognitive impairment (Bennett and Thomas, 2014; Shimada et al., 2014). Therefore, low SES populations are more likely to acquire cognitive impairment.

Our study found that long duration (>9 h) also correlated with an increased risk of cognitive impairment, which is consistent with the findings of Sindi et al. (2018) based on a study of 1,446 individuals. Several studies based on Chinese populations have also confirmed this finding (Xu et al., 2011; Song et al., 2017). This may be due to the fact that prolonged sleep (> 9 h) increases the systemic inflammatory response and elevated levels of inflammatory markers are associated with increased white matter hyper density as well as reduced gray matter volume, which in turn increases the risk of cognitive decline (Irwin et al., 2016).

The relationship between depression and cognitive impairment has been confirmed by numerous studies (Li et al., 2019; Siette et al., 2020). One study showed that the risk of developing dementia in patients with a history of early onset depression was 2.3 times higher than in those with late-onset depression and 3.8 times higher than in those without depression. This may be due to the fact that depressive states activate the hypothalamic-pituitary-adrenal axis, and the hippocampus also causes a reduction in hippocampal volume due to prolonged exposure to glucocorticoids, which in turn contributes to the development of cognitive impairment.

In addition, the present study found an association between marital status and cognitive impairment. This has also been confirmed in a large number of previous studies (Fan et al., 2015; Sundström et al., 2016; Liu et al., 2020). Consistent with the findings of the present study, a study of the risk of dementia in different marital status groups in the United States showed that all unmarried populations, including divorced/separated, widowed, cohabiting, and unmarried populations, had a significantly higher odds of developing dementia than married populations during the study period (Liu et al., 2020). Another national population-based prospective study from Sweden also showed that unmarried people living alone may be at risk for early onset and late-onset dementia (Sundström et al., 2016). A study in Taiwan covering a total of 10,432 residents in 19 counties also confirmed a higher risk of dementia in widows or widowers (Fan et al., 2015). This can be explained by the fact that the loss of a spouse is a stressful event that causes depressed mood, which in turn has been shown to be a risk factor for cognitive impairment disorders. In contrast, living alone and unmarried status triggers loneliness, and feeling lonely is considered a major risk factor for dementia in the elderly independent of vascular disease, depression, and other confounding factors (Holwerda et al., 2014). Marriage, on the other hand, serves as a buffer against the negative consequences of adverse life events, and married individuals usually have more social support to reduce psychological distress, and they also have less anxiety and depression. In addition, studies have confirmed that marital relationships, as one of the most intimate relationships, are one of the best sources of cognitive stimulation (Sjöberg et al., 2020). Thus, marriage can expand the brain’s cognitive reserve to buffer neurological cases from damage and delay the onset of cognitive impairment.

The equity index was calculated to be −0.165 (*p* < 0.001) in this study, indicating that cognitive impairment has an inequity bias toward the poor among older adults in Macau. Decomposition analysis revealed that socioeconomic status contributed the most to the inequity of cognitive impairment. Poor socioeconomic status exacerbated inequity. This was followed by marriage and appetite. This is consistent with the study by Yang et al. (2016). This further illustrates that cognitive impairment inequity is a problem with worldwide prevalence. Cognitive impairment inequity that continues to worsen will be detrimental to overall social welfare. The higher socioeconomic groups may be more advantaged in terms of health because they are less likely to suffer from health injuries due to good working and living conditions, and they are more likely to have better access to health care knowledge and to be more adept at using medical technology to maintain health and prevent cognitive decline due to their educational background, occupational status, and income. In addition, they are more inclined to a healthy lifestyle and social network to delay cognitive decline (Lee et al., 2003). Even when cognitive decline occurs, those with higher socioeconomic status have a better chance than those with lower socioeconomic status to detect the condition and correct the adverse factors in time to avoid further deterioration of cognitive function. This partly confirms the dominance assumption theory (Leung et al., 2017) that cognitive differences between individuals of different socioeconomic status accumulate over time, and therefore the relationship between inequality in cognitive impairment and socioeconomic status is significant in older adults. In this study, a stratified analysis based on gender found an inequity in cognitive impairment in male older adults in favor of the poor, but this inequity was not statistically significant, considering that it may be due to the small sample size of males in this study and the lack of statistical validity.

The present study also has some limitations. First, information on socioeconomic level and lifestyle is mainly self-reported and measured only once, so measurement error is inevitable. Second, we used the MoCA scale to measure cognitive impairment. The MoCA has been used in epidemiological surveys and clinical practice for screening of cognitive impairment and has been shown to have good reliability, so we used this scale in the current investigation, but the use of a single scale may result in misclassification. Therefore, the results should be interpreted with caution in terms of cross-sectional comparisons. Further studies can be conducted using different cognitive measurement tools to compare whether there are differences in the results. Third, this study only examined the socioeconomic inequity of cognitive impairment in Macau, however, some studies have found significant differences in health disparities across different socioeconomic status groups in different regions (De Vogli et al., 2008). Therefore, there is a need to expand the scope of the study for further exploration. Fourth, the subjects in this study were all from nursing homes, which are generally considered to have poorer health status compared to the community, which may overestimate the prevalence of cognitive impairment among older adults in Macau. Fifth, the smaller sample size limited us to conduct more stratified analysis. Sixth, the survey focused on limited factors that might impact, we didn’t assess all the potential key determinants like socioeconomic position in childhood on cognitive aging, so further studies are needed to examine more factors.

## Conclusion

In conclusion, this study found that there is inequality in cognitive impairment among the elderly in Macau, and the occurrence of cognitive impairment is mainly concentrated in the economically disadvantaged population. The contribution of SES to this inequality is significant, and our study has some practical implications. Policy makers and researchers should focus on the needs of the low SES population when making decisions related to cognitive impairment prevention. In addition, reducing the gap between rich and poor at the source, increasing the educational level of low SES populations, and improving the accessibility of health care services for low SES populations will be important to improve the inequity of cognitive impairment.

## Data Availability Statement

The raw data supporting the conclusions of this article will be made available by the authors, without undue reservation.

## Author Contributions

ZZ conducted the data analysis, interpreted the results, drafted, and revised the manuscript. YZ collected the data and edited the manuscript. YB designed the study and edited the manuscript. All authors revised the manuscript and agreed to be accountable for the content of the work, read and approved the final manuscript.

## Conflict of Interest

The authors declare that the research was conducted in the absence of any commercial or financial relationships that could be construed as a potential conflict of interest.

## Publisher’s Note

All claims expressed in this article are solely those of the authors and do not necessarily represent those of their affiliated organizations, or those of the publisher, the editors and the reviewers. Any product that may be evaluated in this article, or claim that may be made by its manufacturer, is not guaranteed or endorsed by the publisher.
