# Supplementary material for: A Role of Socioeconomic Status in Cognitive Impairment Among Older Adults in Macau: A Decomposition Approach
Source: Front Aging Neurosci. 2022 Feb 8;14:804307. doi: 10.3389/fnagi.2022.804307 (PMC8862725; doi:10.3389/fnagi.2022.804307)
Supplement: Supplementary file 1 [file Data_Sheet_1.PDF]

Table S1 Sociodemographic characteristics of subjects according to the region

Variables		Taipa (12)	Coloane (40)	Peninsula (293)
Gender				
	Male	7 (11.67%)	4 (6.67%)	49 (81.67%)*
	Female	5 (1.75%)	36 (12.63%)	244 (85.61%)
Age (years)				
	< 75	5 (3.62%)	14 (10.14%)	119 (86.23%)
	≥75	7 (3.38%)	26 (12.56%)	174 (84.06%)
SES				
	Poor	1 (0.85%)	8 (6.84%)	108 (92.31%)*
	Medium	3 (2.59%)	11 (9.48%)	102 (87.93%)
	Good	8 (7.14%)	21 (18.75%)	83 (74.11%)
Sleep duration（hours）				
	< 7	2 (2.44%)	15 (18.29%)	65 (79.27%)
	7-9	5 (3.21%)	18 (11.54%)	133 (85.26%)
	≥9	5 (4.67%)	7 (6.54%)	95 (88.79%)
Regular exercise				
	No	5 (6.49%)	12 (15.58%)	60 (77.92%)
	Yes	7 (2.61%)	28 (10.45%)	233 (86.94%)
Marital status				
	Others	2 (1.38%)	17 (11.72%)	126 (86.90%)
	Married	10 (5.00%)	23 (11.50%)	167 (83.50%)
Appetite				
	Bad	1 (3.45%)	3 (10.34%)	25 (86.21%)
	Medium	1 (3.03%)	1 (3.03%)	31 (93.94%)
	Good	10 (3.53%)	36 (12.72%)	237 (83.75%)
Tea/coffee drinking				
	No	4 (2.67%)	12 (8.00%)	134 (89.33%)
	Yes	8 (4.10%)	28 (14.36%)	159 (81.54%)
Religious belief				
	No	7 (3.30%)	27 (12.74%)	178 (83.96%)

Yes	5 (3.76%)	13 (9.77%)	115 (86.47%)
Depression			
No	5 (1.93%)	36 (13.90%)	218 (84.17%)*
Yes	7 (8.14%)	4 (4.65%)	75 (87.21%)
Cognitive impairment			
No	8 (66.67%)	36 (90.00%)	148 (50.51%)*
Yes	4 (33.33%)	4 (10.00%)	145 (49.49%)

*P<0.05;

SES: socioeconomic status

Table S2 Univariate analysis of influencing factors of cognitive impairment

Variables		Cognitive impairment	
		No (192)	Yes (153)
Gender			
	Male	34 (56.67%)	26 (43.33%)
	Female	158 (55.44%)	127 (44.56%)
Age (years)			
	< 75	85 (61.59%)	53 (38.41%)
	≥75	107 (51.69%)	100 (48.31%)
SES			
	Poor	48 (41.03%)	69 (58.97%)*
	Medium	63 (54.31%)	53 (45.69%)
	Good	81 (72.32%)	31 (27.68%)
Sleep duration（hours）			
	< 7	51 (62.20%)	31 (37.80%)*
	7-9	96 (61.54%)	60 (38.46%)
	≥9	45 (42.06%)	62 (57.94%)
Regular exercise			
	No	40 (51.95%)	37 (48.05%)
	Yes	152 (56.72%)	116 (43.28%)
Marital status			

Others	66 (45.52%)	79 (54.48%)*
Married	126 (63.00%)	74 (37.00%)
Appetite		
Bad	13 (44.83%)	16 (55.17%)*
Medium	11 (33.33%)	22 (66.67%)
Good	168 (59.36%)	115 (40.64%)
Tea/coffee drinking		
No	73 (48.67%)	77 (51.33%)*
Yes	119 (61.03%)	76 (38.97%)
Religious belief		
No	112 (52.83%)	100 (47.17%)
Yes	80 (60.15%)	53 (39.85%)
Depression		
No	157 (60.62%)	102 (39.38%)*
Yes	35 (40.70%)	51 (59.30%)

*P<0.05;

SES: socioeconomic status

Table S3 Decomposition analysis on the inequality of cognitive impairment in female elderly

	Elasticity	Concentration index	Contribution	Contribution rate
Age (years)				
≥75	0.000	-0.128	<0.000	0.00%
				72.92%
SES				
Poor	1.070	-0.620	-0.663	
Medium	0.549	0.088	0.048	
Sleep duration (h)				1.38%
< 7	-0.256	-0.048	0.012	
≥9	0.187	-0.128	-0.024	
Regular exercise				

Yes	-0.533	-0.008	0.004	-0.51%
Marital status				
Others	0.732	-0.075	-0.055	6.55%
Appetite				9.17%
Bad	0.065	-0.274	-0.018	
Medium	0.335	-0.178	-0.060	
Tea/coffee drinking				
No	0.487	-0.112	-0.055	6.47%
Religious belief				
No	0.217	-0.021	-0.004	0.53%
Depression				
Yes	0.256	-0.115	-0.029	3.49%

SES: socioeconomic status
